# Antifungal activity of silver nanoparticles in combination with ketoconazole against *Malassezia furfur*

**DOI:** 10.1186/s13568-019-0857-7

**Published:** 2019-08-20

**Authors:** Javier Esteban Mussin, María Virginia Roldán, Florencia Rojas, María de los Ángeles Sosa, Nora Pellegri, Gustavo Giusiano

**Affiliations:** 1 Mycology Department, Instituto de Medicina Regional, Universidad Nacional del Nordeste, Consejo Nacional de Investigaciones Científicas y Tecnológicas (CONICET), Av. Las Heras 727, 3500 Resistencia, Chaco Argentina; 2 Laboratorio de Materiales Cerámicos, Instituto de Física Rosario, Universidad Nacional de Rosario, CONICET, Rosario, Argentina

**Keywords:** *Malassezia*, Nanoparticles, Antifungal activity, Synergy

## Abstract

*Malassezia furfur* is lipophilic and lipid-dependent yeast, inhabitant of human skin microbiota associated with several dermal disorders. In recent years, along with the advances in nanotechnology and the incentive to find new antimicrobial drugs, there has been a growing interest in the utilization of nanoparticles for the treatment of skin microbial infections. This work aimed to study the in vitro inhibitory activity of silver nanoparticles (AgNP) against 41 *M. furfur* clinical isolates, visualize the interaction between AgNP-*Malassezia*, evaluate the synergism with ketoconazole (KTZ) and to produce an antimicrobial gel of AgNP–KTZ. The synthesized AgNP were randomly distributed around the yeast surface and showed a fungicidal action with low minimal inhibitory concentration values. AgNP showed no antagonistic effect with KTZ. The broad-spectrum antimicrobial property with fungicidal action of AgNP and its accumulation in affected areas with a sustained release profile, added to the great antifungal activity of KTZ against *Malassezia* infections and other superficial mycoses, allowed us to obtain a gel based on carbopol formulated with AgNP–KTZ with the potential to improve the topical therapy of superficial malasseziosis, reduce the number of applications and, also, prevent the recurrence.

## Introduction

Yeasts of *Malassezia* genus are normal inhabitants of the human skin microbiota and other warm-blooded vertebrates. Since they are unable to synthesize fatty acids, all *Malassezia* species are lipophilic and most of them lipid-dependent, requiring an external source of lipids. For this reason, they prevail in body areas rich in sebaceous glands (Boekhout et al. 2010).

These yeasts are considered to be the etiological agent of pityriasis versicolor and *Malassezia* folliculitis, associated agents in seborrheic dermatitis/dandruff and a contributory factor that exacerbate other skin disorders such as atopic dermatitis, psoriasis, confluent and reticulate papillomatosis, and neonatal pustulosis (Giusiano 2006; Boekhout et al. 2010; Saunders et al. 2012; Rojas et al. 2014; Rudramurthy et al. 2014; Prohic et al. 2016). Cutaneous diseases associated with *Malassezia* are often chronic and recurrent. In these cases, the results of antifungal therapy, both topical and systemic, are not always effective due to high relapse rates (Giusiano et al. 2010; Carrillo-Muñoz et al. 2013; Prohic et al. 2016; Rojas et al. 2016).

Topical antifungal medications are the first-line treatment for *Malassezia* infections, and ketoconazole (KTZ) is one of the most effective antifungal agents. KTZ is a fungistatic imidazole that inhibits the lanosterol 14α-demethylase, an enzyme that regulates the synthesis of ergosterol. The disruption of ergosterol biosynthesis alters cell membrane structure, thus compromising membrane integrity and permeability and consequently interfering with cellular growth and reproduction. KTZ was the first broad-spectrum antifungal used in the treatment of superficial mycoses (Gupta and Foley 2015).

Nanotechnology is an important field of modern research which deals with synthesis and manipulation of structures of matter ranging from approximately 1 to 100 nm in size, commonly called nanomaterials (Liz-Marzán and Kamat 2003; Rao et al. 2004). The noble metal nanoparticles showed unique and considerably different physical and chemical properties compared to their macro scaled counterparts (Feldheim and Foss 2002). As the size of the nanoparticles decreased, their surface-volume ratio and antimicrobial activity increased (Rai et al. 2009; Sharma et al. 2009; Song and Kim 2009; Bera et al. 2014; Ahmed et al. 2016). Silver (Ag) is one of the noble metals with higher antimicrobial activity and lower toxicity for animal cells (ATSDR 1990; Lansdown 2010).

There has been a growing interest in silver nanoparticles (AgNP) over the years due to their potential application in human and animal medicine for treating skin infections including dermatomycosis (Rai et al. 2009; Ge et al. 2014; Aljuffali et al. 2015). Likewise, these particles either alone or in combination with other drugs, would represent a therapeutic alternative against resistant microorganisms, as well as in complications associated with the use of antifungals (Rai et al. 2009, 2012; Bera et al. 2014).

Clinical and Laboratory Standards Institute (CLSI) document M27-A3, describes a broth microdilution method for testing the in vitro antifungal susceptibility for *Candida* species and *Cryptococcus neoformans* for the determination of minimal inhibitory concentrations (MIC) (Clinical and Laboratory Standards Institute 2008). Due to the nutritional requirements of *Malassezia* yeasts, this method is not applicable for this genus. Rojas et al. (2014) proposed a nutritionally supplemented medium to evaluate the in vitro activity of antifungals against some *Malassezia* species.

The aims of this study were to: (a) study the in vitro inhibitory activity of AgNP synthesized against *Malassezia furfur* clinical isolates, (b) evaluate this activity in combination with KTZ, (c) visualize the interaction between AgNP-*Malassezia* and (d) produce and evaluate the activity of an antimicrobial gel of AgNP–KTZ.

## Materials and methods

### Synthesis of silver nanoparticles

AgNP were synthesized by chemical reduction of AgNO_3_ in ethanol, according to Roldán et al. (2008), with some modifications as described as follow. AgNO_3_ (Merck) was dissolved in ethanol absolute (Ciccarelli) under ultrasound stirring. Ethanol was used as solvent and also as a mild reducing agent. On the other hand, the aminosilane [*N*-[3-(trimethoxysilyl) propyl] diethylenetriamine] (ATS, Aldrich) was dissolved in ethanol absolute under N_2_ atmosphere and magnetic stirring. Then, both solutions were mixed and homogenized obtaining a final solution of 12 mM of AgNO_3_ and 0.197 M of ATS. This solution was placed at a temperature bath at 40 °C under N_2_ atmosphere for 4 h. The color of the solution changed from uncolored to bright yellow.

### Characterization of silver nanoparticles

It was carried out by UV–Vis absorption spectroscopy and transmission electron microscopy (TEM).

Optical characterization of colloidal suspensions was performed by UV–Vis absorption spectroscopy, using UV/Vis spectrophotometer (Jasco V-530). UV–Vis spectra were acquired by employing ethanol as reference. The colloidal stability of AgNP over time was evaluated by visual observation and UV–Vis spectroscopy.

TEM images were acquired with a Phillips 100 keV. Samples were prepared by dropping the nanoparticles suspension over a carbon coated TEM grid. Several TEM images were processed with ImageJ free software to estimate mean size and standard deviation.

### Microorganisms

A total of 40 *M. furfur* isolates were studied. Isolates were obtained from human clinical samples with diagnosis of pityriasis versicolor, seborrhoeic dermatitis/dandruff and atopic dermatitis. All *Malassezia* yeasts were deposited in the culture collection of Mycology Department, Instituto de Medicina Regional (IMR), Universidad Nacional del Nordeste (UNNE), Argentina. Identification was performed by polymerase chain reaction-restriction fragment length polymorphism (PCR–RFLP) (Mirhendi et al. 2005; Sosa et al. 2013). In addition, the reference strain *M. furfur* CBS 7019 was included.

Isolates were sub-cultured for 72 h onto modified Dixon Agar at 32 °C before antifungal susceptibility testing.

### Minimum inhibitory concentration (MIC)

In order to evaluate the inhibitory activity of synthesized AgNP and KTZ (Sigma-Aldrich, Buenos Aires, Argentina), MIC were determined by broth microdilution method in accordance with CLSI M27-A3 document (Clinical and Laboratory Standards Institute 2008) with modifications proposed by Rojas et al. (2014).

All inoculum suspensions were prepared in sterile saline solution and turbidity was adjusted to a 1 McFarland scale by densitometer (DEN-1 densitometer, Biosan). This inoculum was diluted 1:100 in supplemented RPMI medium to achieve a final concentration of 0.5–2.5 × 10^5^ CFU/mL.

AgNP and KTZ solutions were prepared using dimethyl sulfoxide (DMSO) as solvent (final concentration ≤ 1%) and RPMI medium as diluents. A twofold dilution serial of the drugs was performed to obtain a final concentration range from 4 to 0.008 mg/L. The microtiter plates with 96 U wells (Greiner bio-One, Buenos Aires, Argentina) were incubated for 3 days at 32 °C.

MIC for AgNP solution was determined by visual reading of growth inhibition at two endpoints: MIC-2 as the lowest concentration capable of inhibiting ≥ 50% growth as compared with the AgNP-free growth-control well and MIC-0 as the lowest concentration that completely inhibit yeast growth. For KTZ, the MIC endpoint was MIC-2 (Clinical and Laboratory Standards Institute 2008).

### Visualization of interaction AgNP-*Malassezia*

To observe the yeasts after treatment with AgNP, 10 µL were taken from the well corresponding to a MIC-2 for *M. furfur* CBS 7019 and placed on a clean and sterile glass surface. After drying at 35 °C under sterile conditions, cells were fixed with a solution of formaldehyde–alcohol–acetic acid (FAA) for 24 h. Subsequently, dehydration was carried out using an ethanol gradient, critical point drying in CO_2_ and gold coating. Samples were examined under a Jeol 5800 LV (Tokyo, Japan) scanning electron microscope (SEM) at Servicio de Microscopía Electrónica (Universidad Nacional del Nordeste, Argentina).

### Minimum fungicidal concentration (MFC)

The MFC of AgNP solution was determined following procedures of Cantón et al. (2003) with modifications. After the MIC was read, the content of each well was homogenized with a micropipette and the entire volume (200 µL) of these wells was subcultured onto modified Dixon agar plates of 90 × 15 mm. Aliquots were deposited as a spot onto the agar and after the plate was dry, streaking was performed. All plates were incubated at 32 °C for 72 h. The MFC was defined as the lowest drug concentration at which no colonies were observed (99.9% killing activity).

Since the AgNP mode of action is still unknown and there is no consensus on the endpoint reading, MIC-2, MIC-0 and the MFC were determined.

MFC/MIC ratios were calculated for each isolate using MIC-0 value. By extrapolation from the conventional definition used for bacterial testing, in this work, a compound was considered fungicidal when the MFC/MIC ratio was ≤ 4 and fungistatic when the MFC/MIC ratio was > 4 (Hazen 1998; Pfaller et al. 2004; Meletiadis et al. 2007).

MFC/MIC ratio allows us to determine if the isolate was tolerant to the compound. Tolerance occurs when a fungi is inhibited but not killed by an antifungal agent that normally is considered fungicidal, defined by a MFC/MIC ratio > 32 (Pfaller et al. 2004).

All assays were performed in duplicate. The range, geometric mean, mode, median, standard deviation, MIC_50_ and MIC_90_ values defined as the lowest concentration at which 90% and 50% of all isolates tested were inhibited, were also obtained. Statistical analysis was carried out using InfoStat software provided by Cátedra de Estadística y Biometría, Facultad de Ciencias Agropecuarias, Universidad Nacional de Córdoba, Argentina.

### Synergism

Checkerboard microdilution method was used to evaluate the synergistic antifungal effect as a consequence of KTZ–AgNP interaction (Odds 2003). This assay was performed in 96-well microplates in which each row and each column contained twofold serial dilutions of KTZ and AgNP respectively, at concentrations around and below its MIC, reaching a unique combination of the two substances in each well. To evaluate combination of drugs against *Malassezia*, yeast inoculum, culture medium, temperature and incubation time, in identical conditions used to evaluate the in vitro inhibitory activity, were used.

Interactions were quantitatively evaluated by determining the fractional inhibitory concentration index (FICi) according to the formula (Meletiadis et al. 2002; Odds 2003):

$$FICi = FIC_{KTZ} + FIC_{AgNP} = \frac{{MIC_{KTZ} \;in\;combination}}{{MIC_{KTZ} \;alone}} + \frac{{MIC_{AgNP} \;in\;combination}}{{MIC_{AgNP} \;alone}}$$

FICi values were interpreted accordingly to Odds (2003) as: synergism (FICi ≤ 0.5), no interaction (FICi > 0.5–4.0) and antagonism (FICi > 4.0).

The FICi was determined for all the possible combinations of different concentrations for each isolate.

### Gels preparation

Four formulations of gels based on carbopol 940 (Merck) at 1.5% (w/w) were prepared. The formulation A contained a final AgNP concentration of 0.03 mg/g; formulation B contained a final KTZ concentration of 0.03 mg/g; formulation C contained a final AgNP and KTZ concentration of 0.03 mg/g and 0.03 mg/g, respectively. Formulation D without drugs was used as control.

Required quantity of carbopol 940 was weighed and dispersed slowly in the sterile purified water with continuous agitation. After that, the polymer was allowed to stand for 24 h until it was completely wetted. The drug corresponding to each formulation was added with continuous stirring till it was completely dispersed. Finally, the pH of the gel was adjusted to pH 7 using triethanolamine. The prepared gels were packaged under sterile conditions and stored in dark and cool place until time of use.

### In vitro antifungal activity of gels

In order to evaluate the in vitro antifungal activity of the gel formulations by agar well diffusion method against *M. furfur* CBS 7019, 0.5 g of each gel containing 15 µg of each compound, was placed in a 6 mm diameter well separated by a distance of 2.5 cm. The inoculum size, culture medium, temperature and incubation time used were according to Rojas et al. methodology (Rojas et al. 2016). The diameter of the inhibition zone was measured after incubation.

To observe whether the medium of support (carbopol 940) modified the drugs activity, the disk diffusion assay against *M. furfur* CBS 7019 according to Rojas et al. (2016) with modifications (Svetaz et al. 2016) was performed. Sterile Schleicher & Schuell 9 mm paper disks were embedded with 15 µg KTZ; others with 15 µg AgNP and a third disk with a mixture of both drugs (15 µg KTZ + 15 µg AgNP). A fourth disk without drugs was used as the control. Plates were incubated at 32 °C for 72 h. The diameter of the inhibition zone around each disk was measured after incubation.

## Results

**Fig. 1:**
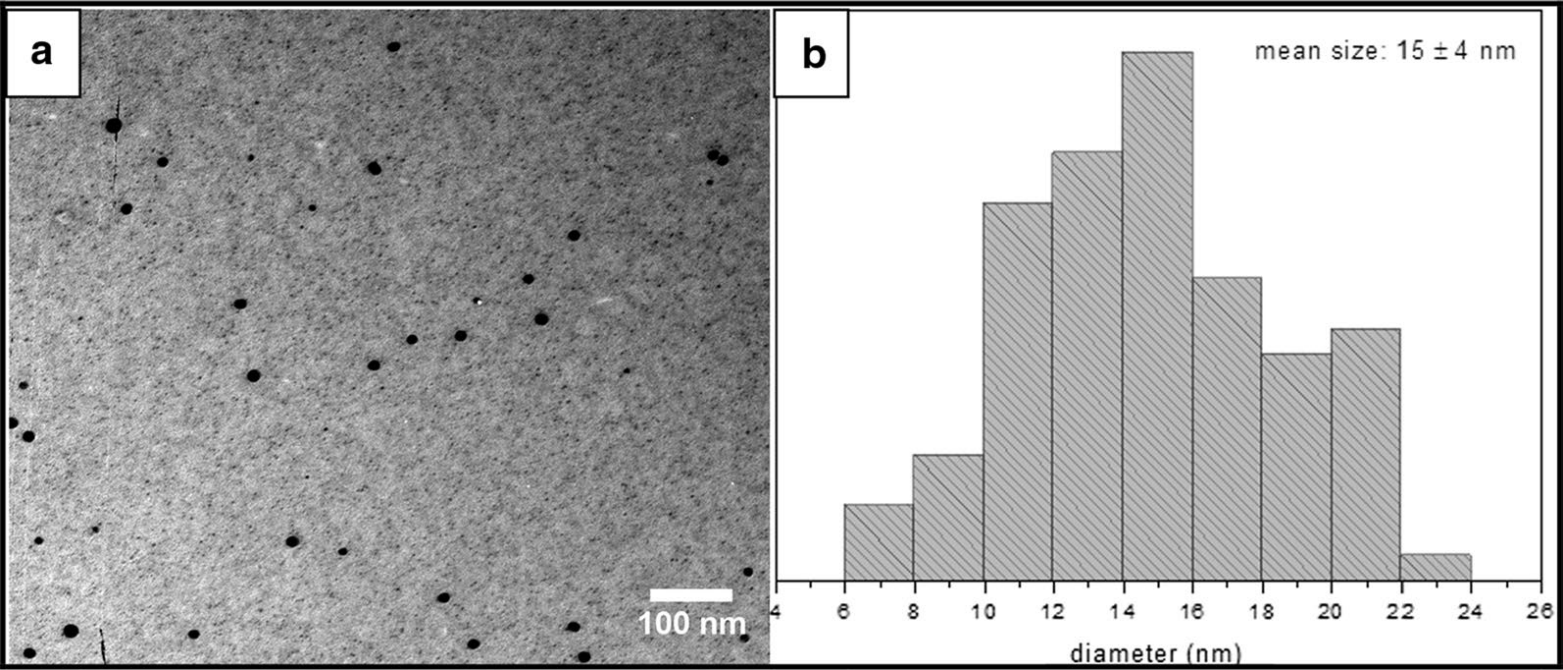
**a** Silver nanoparticles (AgNP) image acquired by transmission electron microscopy (TEM). **b** Size distribution calculated from several TEM images

The reduction of silver ions in different solutions generally yields colloidal silver with particle diameter of several nanometers and the yellow color is a result of the presence of few nanometers radius Ag nanospheres (Kreibig and Vollmer 1995). In this work, the yellow color of the colloidal silver was evidenced. UV–Vis spectrum of synthesized colloidal nanoparticles showed a maximum absorption band at 408 nm, corresponding to the Local Surface Plasmon Resonance (LSPR) absorption typical of particles with nanometric size. AgNP were stable as colloids for at least 20 months without precipitation and color changes. TEM images showed spherical nanoparticles of 15 ± 4 nm diameter, measuring 100 particles from several images (Fig. 1). None agglomerated particles were observed.

Antifungal activity of AgNP and KTZ against *M. furfur* evaluated by broth microdilution method showed in vitro inhibitory activity against all isolates of *M. furfur* with MIC-2 values less to 0.5 mg/L. Also, KTZ showed values of geometric mean, mode, MIC_50_ and MIC_90_ lower than AgNP. However, the range obtained with AgNP was lower than KTZ (Table 1).

**Table 1:** MFC, MIC-2 and MIC-0 ranges, geometric mean (Gm), mode, median, standard deviation (Sd) and MIC_50_ and MIC_90_ obtained for 41 *M. furfur* isolates

	Range	Gm	Mode	Median	Sd	MIC_50_	MIC_90_
AgNP MIC-2	0.06–0.25	0.11	0.12	0.12	0.06	0.12	0.25
AgNP MIC-0	0.12–2.00	0.51	1	0.5	0.54	0.50	1
AgNP MFC	0.25–2.00	0.74	1	1	0.69	–	–
AgNP MFC/MIC	1.00–2.00	1.61	2.00	2.00	0.47	–	–
KTZ	0.016–0.50	0.04	0.03	0.03	0.09	0.03	0.06

All MIC and MFC data are expressed in terms of mg/L

To date, there is no consensus on the MIC reading endpoint for AgNP against *Malassezia*, consequently, MIC-0 and MIC-2 were determined. Comparing MIC-0 and MIC-2 data obtained with MFC for AgNP, MIC-0 showed a wide dispersion of values but with range, geometric mean, mode, median and standard deviation more similar to MFC (Table 1). In addition, 56.52% of MIC-2 values showed a difference of 3 or more dilutions with respect to MFC. In contrast, differences ≤ 1 dilution with respect to MFC were obtained in 100% of MIC-0. Therefore, the MIC-0 values were more consistent with the MFC values.

Since it is unknown if AgNP has fungicidal or fungistatic activity, the MFC/MIC ratios were calculated. Table 1 show that all ratios obtained were less than 4, suggesting that AgNP has fungicidal activity against *M. furfur*. Furthermore, no tolerance effect was evident.

**Fig. 2:**
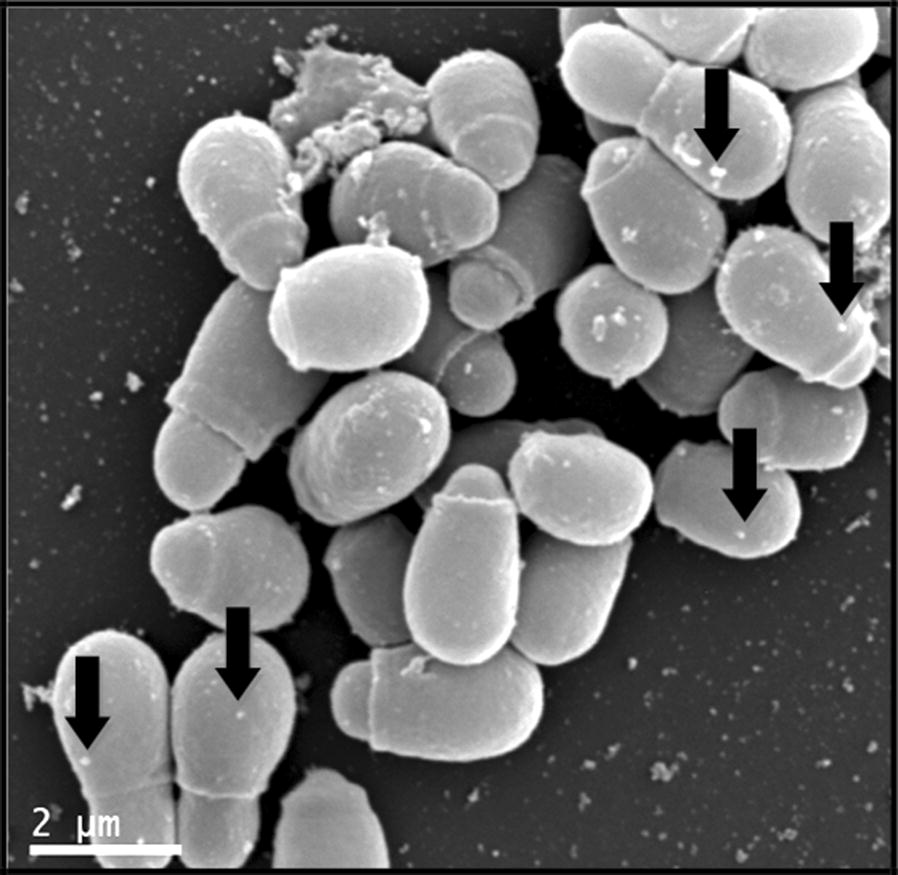
Visualization of interaction between AgNP and *M. furfur* CBS 7019 by SEM. The black arrows show the interaction of AgNP on the surface of *Malassezia* yeasts

Applying SEM, micromorphology of *M. furfur* yeasts and the interaction AgNP-*Malassezia* could be observed as shown in Fig. 2. Nanoparticles adhere to the fungal cell wall with a non-specific distribution.

Checkerboard assay allowed to evaluate the effect of the combination of AgNP with KTZ against *M. furfur*. FICi values obtained are shown in Table 2. Interaction varied among different strains tested, 82.92% showed no-interaction effect (FICi > 0.5–4.0), while only 17.08% showed synergistic effect (FICi ≤ 0.5).

**Table 2:** Values obtained of interaction between AgNP and KTZ against *M. furfur* using checkerboard assay

FICi	Interpretation	n	Percentage (%)
0.375	Synergism	2	4.88
0.50	Synergism	5	12.20
0.75	No interaction	26	63.41
1.00	No interaction	8	19.51
Total		41	100

**Fig. 3:**
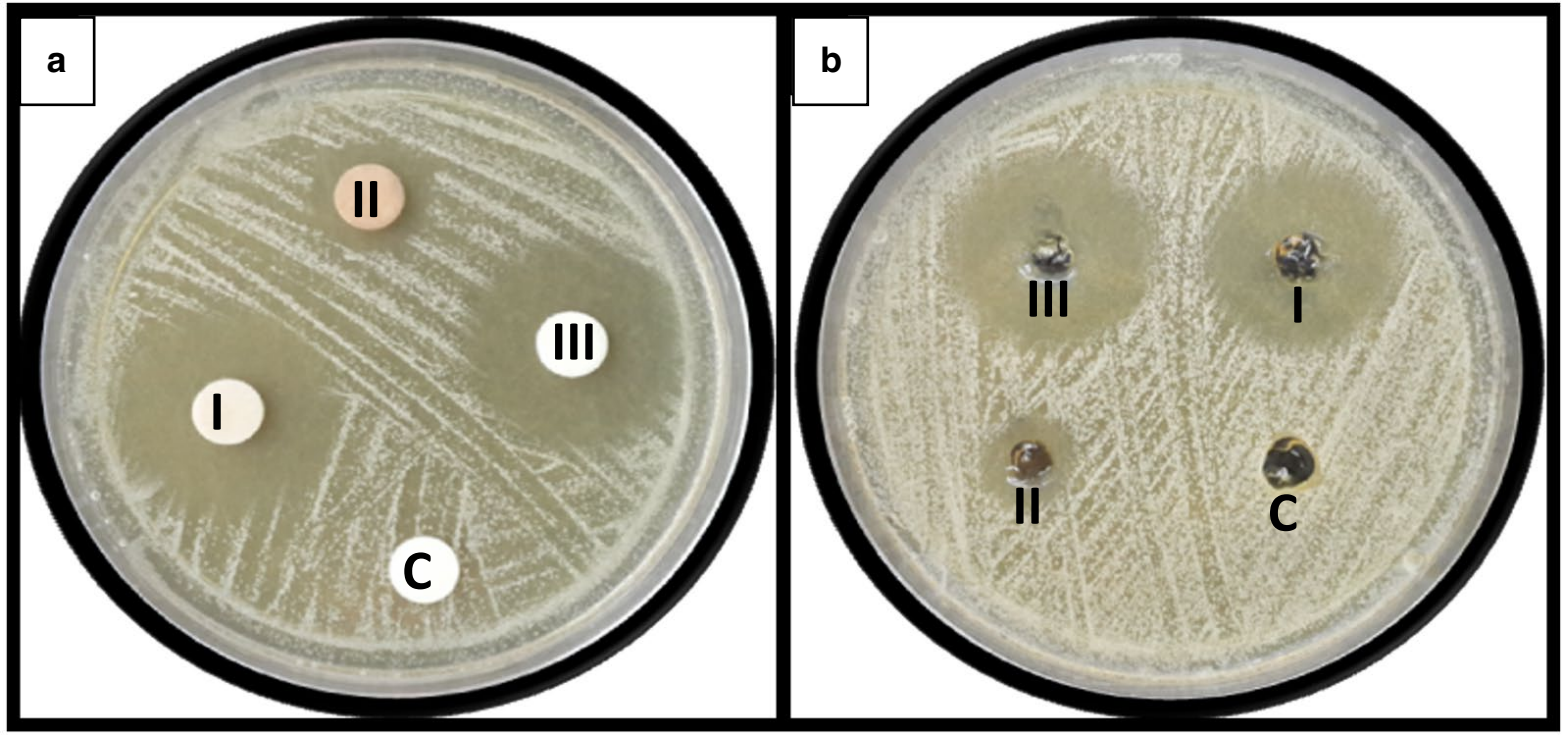
Evaluation of the antifungal activity against *M. furfur* CBS 7019 by **a** disk diffusion and **b** agar well diffusion assay. I: KTZ; II: AgNP; III: KTZ + AgNP; C: control

Inhibition zones diameters obtained by both diffusion assays for AgNP, KTZ and AgNP–KTZ against *M. furfur* CBS 7019 are summarized in Table 3 and can be observed in Fig. 3. AgNP–KTZ association showed a little increase in the inhibition zone compared to KTZ alone. However, this increase was higher using AgNP–KTZ in gel formulation. The drug-free gel formulation showed no inhibition zone. Therefore, carbopol did not negatively modify the antifungal activity of drugs.

**Table 3:** Inhibition zone diameters (mm) of the different drugs against *M. furfur* CBS 7019

	Disk	Gel
Control	9	6
AgNP	15	13
KTZ	27	24
KTZ + AgNP	28	28

## Discussion

In recent years, along with the advances in nanotechnology and the incentive to find new antimicrobial drugs, there has been a growing interest in the utilization of nanoparticles for the treatment of skin microbial infections (Rai et al. 2014; Aljuffali et al. 2015). The antimicrobial properties of silver have been recognized and used as a standard treatment for bacterial skin infections caused by *Staphylococcus aureus* and *Pseudomonas aeruginosa* (Aljuffali et al. 2015). On the other hand, AgNP have shown a broad-spectrum antimicrobial activity including fungal agents of opportunistic infections (Rai et al. 2014) such us *Candida albicans*, *C. tropicalis*, *C. parapsilosis*, *C. glabrata* (Panácek et al. 2009; Rahisuddin et al. 2015), *Trichophyton rubrum* (Kim et al. 2008; Pereira et al. 2014), *Trichosporon asahii* (Xia et al. 2014), *Aspergillus niger*, *Rhizoctonia solani*, *Curvularia lunata*, *Colletotrichum* sp. and *Fusarium* sp. (Bera et al. 2014; Balakumaran et al. 2015). However, some considerations must be taken into account in those reports. The synthesis methods used differs as well as the size and structure of the AgNP. Furthermore, in most of them, only a single isolate was used as a representative of the species.

In our work, AgNP were synthesized by a low cost simple continuous media (Roldán et al. 2008). ATS was used as a surface modifier and colloidal stabilizer, inhibiting the growth and avoiding agglomeration of reduced Ag^0^, influencing the nanoparticles morphology and size. In addition, the use of aminosilanes has been shown to have a good biocompatibility for nanoparticles of different compositions (Zhu et al. 2012; Datta et al. 2017). Therefore, the method applied in synthesizing AgNP allowed us to obtain stable particles with suitable size and shape, as well as characteristics associated with a consistent antimicrobial activity (Rai et al. 2014).

Ketoconazole (KTZ) was reported as one of the most active drugs against *M. furfur*, with low MIC and low variation in susceptibility values among different isolates. In contrast, high MIC and wide MIC ranges with fluconazole, miconazole and amphotericin B were reported (Garau et al. 2003; Velegraki et al. 2004; Miranda et al. 2007; Carrillo-Muñoz et al. 2013; Rojas et al. 2014). In this work, KTZ also showed a great inhibitory activity against *M. furfur*, with similar values than those reported in other studies (Carrillo-Muñoz et al. 2013; Rojas et al. 2014, 2016). On the other hand, the in vitro inhibitory activity of AgNP was similar to KTZ, showing an even more restricted MIC-2 range (Table 1). Also, 90% of all clinical isolates were inhibited at AgNP concentrations ≤ 0.25 and 1 mg/L when MIC-2 and MIC-0 were used as the reading endpoint, respectively. These results show the strong antifungal activity of synthesized nanoparticles against these lipodependent yeasts.

The AgNP activity was tested against *Malassezia* yeasts using two endpoints. The application of a less rigorous endpoint, such as MIC-2, has been shown to consistently represent the in vitro activity of some compounds (Clinical and Laboratory Standards Institute 2008); in addition, it sometimes provides a better correlation with the in vivo behavior and with other measurements of antifungal activity such as the MFC (Klepser et al. 1998; Ernst et al. 2002). According to the data obtained, MIC-2 values showed a lower dispersion than MIC-0 (Table 1). However, MIC-0 showed values of range, geometric mean, mode, median and standard deviation more similar to MFC, suggesting that MIC-0 is a better reading endpoint. On the other hand, according to considerations proposed in other studies regarding the MFC/MIC ratios (Hazen 1998; Pfaller et al. 2004; Meletiadis et al. 2007), it could be considered that AgNP has fungicidal action against *M. furfur*. Also, none of the isolates showed tolerance effect. Consequently, MIC-0 proved to be the best MIC reading endpoint for this compound against *M. furfur* despite having a wider range, since MIC-2 is used for drugs with fungistatic action. However, it is necessary to correlate these values with other studies like time-kill assays to confirm the fungicidal action.

In applying SEM, it was observed that the nanoparticles adhered to the yeasts cell wall and showed a non-specific distribution, similar to that reported in bacteria and *C. albicans* (Chwalibog et al. 2010; Le et al. 2012; Vazquez-Muñoz et al. 2014; Lara et al. 2015). It is believed that AgNP attach and anchor to the surface of the fungus and produce an increase of reactive oxygen species (ROS). This interaction causes structural changes and damage, markedly disturbing vital cell functions, such as permeability and the membrane potential, forming pores causing ion leakage and other materials, depressing the activity of respiratory chain enzymes and, finally, leading to cell death (Hwang et al. 2012; Vazquez-Muñoz et al. 2014; Lara et al. 2015). Also, it was shown that the accumulation of extracellular AgNP suggests a dynamic release of silver ions (Ag^+^) by adjacent AgNP that actively penetrate the cell and lead to the intracellular biosynthesis of AgNP. The interaction of AgNP with phosphorus- or sulphur-containing compounds as DNA and thiol groups of proteins can cause further damage of yeasts by inhibition of DNA replication and protein inactivation. Furthermore, the gradual release of Ag^+^ by AgNP could have special relevance, as they may act as a reservoir increasing the duration of the antimicrobial effects (Le et al. 2012; Rai et al. 2012; Vazquez-Muñoz et al. 2014).

Topical antifungal medications are the first-line treatment for superficial malasseziosis and KTZ is one of the most widely prescribed. However, treatment of fungal diseases such as pityriasis versicolor is not always effective, have a high recurrence rate and patient application compliance may be affected by multiple or laborious applications, especially in cases where large body areas are affected (Gupta and Foley 2015). On the other hand, the accumulation of AgNP in certain skin areas allow a sustained release of Ag at the infection site over a period of days or even weeks and with more concentration in hair follicles and sebaceous glands zones, where *Malassezia* is more frequently located (Boekhout et al. 2010; Aljuffali et al. 2015). Topical administrations of AgNP can persist more than 10 days on the skin, a more effective treatment can be obtained by reducing the number of applications that lead to greater patient compliance and, also, to avoid possible adverse effects associated with a frequent use. Moreover, the use of AgNP in association with KTZ would allow us to take advantage of the great antifungal activity of KTZ against *Malassezia*, even in other superficial mycoses, and the AgNP would broaden the antimicrobial spectrum and reduce the number of applications.

There are many studies about the combined use of AgNP with antibiotics; however, studies about the antifungal effect of AgNP in combination with clinically-used antifungal drugs are limited (Rai et al. 2014). In the present study, AgNP and KTZ acted independently against most strains when used in combination. None antagonistic effect was detected, but synergistic was observed in 17.08% of the isolates (Table 2). The synergistic effect observed in some cases may be due to the fact that the AgNP acted as a carrier of KTZ, facilitating penetration into yeasts (Durán et al. 2011). These results highlight the importance of testing several isolates of a species to evaluate the activity of antimicrobial drugs, especially combinations of them. Conclusive synergy results should not be obtained using a single strain.

In this work, based on the observed KTZ and AgNP capacities, we seek to take advantage of the benefits of the combined use against *Malassezia* to obtain a carbopol hydrogel containing AgNP and KTZ. Carbopol is a polymer of acrylic acid cross-linked with polyalkenyl ethers or divinyl glycol and is one of the most common thickening agents used by the pharmaceutical and cosmetic industry. Their rheological properties in the aqueous medium are well known and it is essentially a non-toxic and non-irritating material for topical use (Das et al. 2013). There is concern that the silver ions applied to the skin could be absorbed by the systemic circulation in significant amounts, which would increase the risk of poisoning. However, several toxicity studies have shown that AgNP is completely safe for topical administration in concentrations sufficient to inhibit microbial growth (Alt et al. 2004; Jain et al. 2009; Jung et al. 2009; Brandt et al. 2012; Aljuffali et al. 2015). The inhibition zones obtained by both diffusion methods were concordant with the synergy assays. The combined use of AgNP and KTZ showed a small increase in the inhibition zones, being slightly higher when the gel formulation was tested (Table 3 and Fig. 3). It was confirmed that the carbopol formulations obtained do not decrease the antifungal activity of the drugs tested. The drug-free carbopol gel proved to be inactive and a good support medium for both compounds.

As *Malassezia* form part of the skin microbiota, recurrence is particularly difficult to eradicate (Gupta and Foley 2015). Therefore, combination treatment of KTZ with AgNP may be promising in preventing the relapse. Also, antifungal clinical agents are limited and there are few topical medications with fungicidal activity, such as the synthesized AgNP in this work against *M. furfur*. The broad-spectrum of action of AgNP and its easy incorporation into a diversity of support medium such as creams, gels, shampoos, lotions, etc., opens the door to develop new formulations with potential clinical use (Rai et al. 2009; Mijnendonckx et al. 2013). Due to its sustained release profile, accumulation in affected areas, hydro and liposoluble nature, stability and broad-spectrum antimicrobial property, association with KTZ can improve the topical therapy of superficial malasseziosis and, also, prevent its recurrence.

## Data Availability

All relevant data are included in this manuscript.
